# Peer Observations: Enhancing Bedside Clinical Teaching Behaviors

**DOI:** 10.7759/cureus.7076

**Published:** 2020-02-22

**Authors:** Kimberly Pedram, Michelle N Brooks, Carolyn Marcelo, Nargiza Kurbanova, Laura Paletta-Hobbs, Adam M Garber, Alice Wong, Rehan Qayyum

**Affiliations:** 1 Internal Medicine, Division of Hospital Medicine, Virginia Commonwealth University School of Medicine, Richmond, USA; 2 Internal Medicine, Virginia Commonwealth University School of Medicine, Richmond, USA

**Keywords:** bedside teaching, teaching feedback, peer observation, resident education, medical education

## Abstract

Background

Medical training relies on direct observations and formative feedback. After residency graduation, opportunities to receive feedback on clinical teaching diminish. Although feedback through learner evaluations is common, these evaluations can be untimely, non-specific, and potentially biased. On the other hand, peer feedback in a small group setting or lecture format has been shown to be beneficial to teaching behaviors, however, little is known if peer observation using a standardized tool followed by feedback results in improved teaching behaviors. Therefore, the objective of this study was to examine if feedback after peer observation results in improved inpatient teaching behaviors.

Methods

This study was conducted at a tertiary care hospital. Academic hospitalists in the Division of Hospital Medicine developed a standardized 28-item peer observation tool based on the Stanford Faculty Development Program to observe their peers during bedside teaching rounds and provide timely feedback after observation. The tool focused on five teaching domains (learning climate, control of session, promotion of understanding and retention, evaluation, and feedback) relevant to the inpatient teaching environment. Teaching hospitalists were observed at the beginning of a two-week teaching rotation, given feedback, and then observed at the end of the rotation. Furthermore, we utilized a post-observation survey to assess the teaching and observing hospitalists’ comfort with observation and the usefulness of the feedback. We used mixed linear models with crossed design to account for correlations between the observations. Models were adjusted for gender, age, and years of experience. We tested the internal validity of the instrument with Cronbach’s alpha.

Results

Seventy (range: one to four observations per faculty) observations were performed involving 27 teaching attendings. A high proportion of teachers were comfortable with the observation (79%) and found the feedback helpful (92%), and useful for their own teaching (88%). Mean scores in teaching behavior domains ranged from 2.1 to 2.7. In unadjusted and adjusted analysis, each teaching observation was followed by higher scores in learning climate (adjusted improvement = 0.09; 95% CI = 0.02-0.15; p = 0.007) and promotion of understanding and retention (adjusted improvement = 0.09; 95% CI = 0.02-0.17; p = 0.01). The standardized observation tool had Cronbach’s alpha of 0.81 showing high internal validity.

Conclusions

Peer observation of bedside teaching followed by feedback using a standardized tool is feasible and results in measured improvements in desirable teaching behaviors. The success of this approach resulted in the expansion of peer observation to other Divisions within the Department of Internal Medicine at our Institution.

## Introduction

Academic hospitalists spend significant time providing education to learners as teaching attendings. However, hospitalists have varying degrees of experience and training on being an effective clinical educator [1,2]. Teaching attendings receive feedback through learner evaluations, which has been shown to improve teaching effectiveness, but to provide anonymity to the learner, these evaluations are usually aggregated and given to the attending months later, limiting timely improvements [3]. In addition, learners may lack the framework to give effective feedback on teaching and may base evaluations on a variety of factors, such as a desire to achieve a good grade [4]. Peer observation with feedback is a solution to the drawbacks of learner evaluation of teaching attendings. Peer observation of teaching behaviors encourages reflection by both the observer and the teaching attending being observed, leading to increased confidence and performance [5,6]. In addition, peer observation with feedback may positively change bedside teaching style and add new teaching behaviors [7,8].

While studies have evaluated peer observation of teaching skills in lecture or small group settings, there is a paucity of studies examining the effect of feedback provided by peers observing the teacher during bedside rounds [9,10]. The Stanford Faculty Development Program (SFDP) describes seven domains of effective clinical teaching: learning climate, control of teaching session, communication of goals, promotion of understanding and retention, evaluation, feedback, and promotion of self-directed learning [11]. Institutions have used these domains to create evaluation tools assessing the effectiveness of clinical teaching [4,6,12]. A more recent study utilized peer observations of teaching for feedback and then analyzed self-reported changes in teaching behaviors by faculty [5]. None of these studies directly evaluated if feedback from peer observations leads to improvements in teaching behaviors or skill development over time. Thus, whether observation-based peer feedback results in objective and observable improvement in teaching behaviors is not known.

Therefore, the primary objective of this study was to examine if peer observation followed by directed feedback improves teaching behaviors in an inpatient teaching setting. Secondary objectives were to determine if the teaching and observing hospitalists found peer observation followed by feedback useful and if the comfort level of the teaching and observing hospitalists increased following peer observation.

## Materials and methods

The study was conducted at a tertiary care academic medical center. Academic hospitalists, while attending on the general internal medicine ward or consult teams, were observed during bedside teaching rounds by a group of their peers. The observations were conducted over an eight-month period on various weekday mornings from October 2017 to June 2018. Each teaching team consisted of one resident, two interns, and one to two third-year medical students. Each teaching attending was observed twice, once at the beginning of the inpatient rotation and then near the end of that inpatient rotation. The time interval between the two observations was at least one week to allow the incorporation of peer feedback in bedside teaching. Teaching attendings were observed by peers using a standardized tool. The study was deemed exempt by the Institutional Review Board of the Virginia Commonwealth University (approval number: HM20013356).

Peer observation process

Teacher-observer pairings were determined by peer observer availability. A teaching hospitalist could be observed more than twice if that hospitalist was on teaching service for more than one teaching block and peer-observations were logistically possible. No teaching attending had the same observer for consecutive observations. All members of the teaching team were notified in advance about the peer observation and that the observer would be focusing on the teaching attending and not on the learners. After completion of each observation, the observer provided the teaching attending with a written copy of the completed peer observation tool and verbal feedback. Peer observation tools remained confidential; an observer was not allowed to review the prior performance of the teaching attending during earlier observations.

Development of the peer observation tool

A standardized peer observation tool was developed using the SFDP seven-category educational framework allowing for consistent evaluation of teaching skills [6,11,13,14]. The peer observation tool was created focusing on behaviors which could be objectively evaluated during bedside teaching in the inpatient setting. This tool incorporated five of the seven SFDP categories based on relevance to the inpatient teaching environment and had 28 items (learning climate: eight items, control of session: four items, promotion of understanding and retention: eight items, evaluation: five items, and feedback: three items). The tool was scored on a Likert scale using the frequency of an observed behavior (not observed, sometimes observed, and consistently observed). In addition, each item also had comment boxes for formative feedback. The tool included two summary questions completed by the observer asking what the teacher did particularly well and what suggestions observer had for change if any. The peer observation tool was piloted and feedback from the pilot was used to create the final tool.

Surveys

At the start of the study, all participating teaching hospitalists provided demographic information. After completion of an observation, teaching attendings were asked to complete a short survey in which they reported their comfort level with the observation and the usefulness of the feedback they received. Likewise, peer observers were asked to complete a similar survey after observations, reporting their comfort level with the observation and with providing feedback.

Data analysis

Descriptive statistics were reported as mean and standard deviation (SD) for continuous variables and as frequencies for categorical variables. A P-value of <0.05 was considered statistically significant. P-values for continuous variables were calculated using t-test and for categorical variables using Chi-square and Fisher’s exact test as appropriate. Correlations between domains were calculated using Pearson’s correlation. We used mixed-linear growth curve models to examine the association of teaching skills with the feedback. Mixed models were used with crossed design to account for three types of correlations between the observations: one for the same teaching attending on separate observations; second, for the same peer-observer scoring the different teaching attending; and third, for the same teaching attending scored by different peer-observer. Another advantage of using mixed models is that the baseline observation of each participant acts as its own control allowing determination of each individual’s improvement trajectory and then mean trajectory for the whole group. All models were adjusted for gender, age, and years as an attending for both teaching attendings and observers. To examine the internal validity of the instrument, we used Cronbach’s alpha. All statistical analyses were performed using the Stata/MP version 14 for Windows (StataCorp LP, College Station, TX).

## Results

Effect of peer observations on improvement in teaching skills

Seventy peer observations were performed involving 27 teaching attendings. Attendings were observed anywhere from one to four times during the study. Of the 27 teaching attendings included in this study, the majority were females (70%), Caucasian (67%), and graduates of United States medical schools (85%) with mean age of 37.7 (5.1) years and years as attendings of 6.8 (4.4) years, and five (18.5%) were at or above the rank of associate professor. Each peer observation lasted an average of 73 (17.3) minutes. Mean scores of teaching behavior domains ranged from 2.1 to 2.7 (Table 1) and all domains were significantly correlated with each other (correlation between any two domains ranged from 0.3 to 0.7 with significant P-values; Table 1) with Cronbach’s alpha of 0.81 for the tool.

**Table 1:** Correlation between the domains of the peer observation tool Pearson’s correlation with p-values

	Learning	Control	Promotion	Evaluation	Feedback	Mean (SD)
Learning climate domain	1.00					2.6 (0.32)
Control of session domain	0.39 (<0.001)	1.00				2.7 (0.33)
Promotion of understanding and retention domain	0.60 (<0.001)	0.55 (<0.001)	1.00			2.1 (0.55)
Evaluation domain	0.31 (<0.001)	0.42 (<0.001)	0.70 (<0.001)	1.00		2.3 (0.54)
Feedback domain	0.32 (0.006)	0.27 (<0.001)	0.42 (<0.001)	0.30 (<0.001)	1.00	2.7 (0.37)

**Figure 1:**
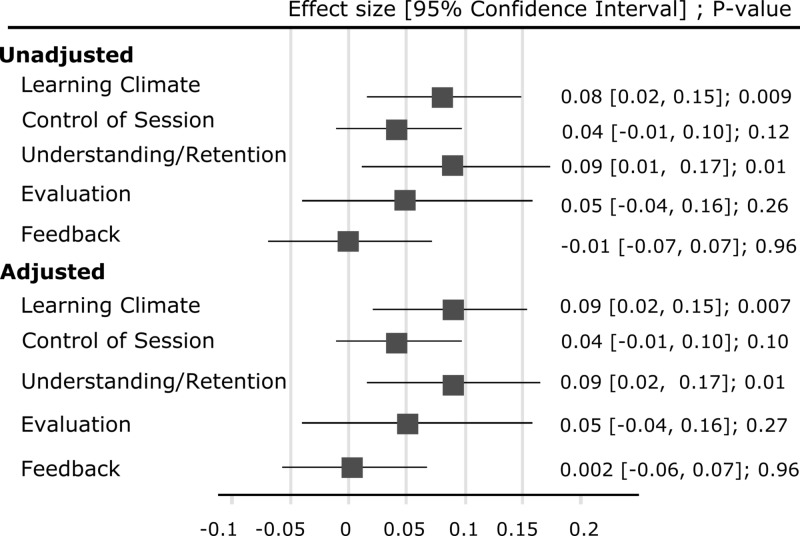
Forest plot of mixed linear growth curve models without and with adjustments *Adjusted analyses were performed including age, gender, years as attending and as covariates in regression models.

In unadjusted analysis, each peer observation was followed by 8% increase in learning climate domain (0.08, 95% CI: 0.02-015, P = 0.009) and 9% increase in promotion of understanding and retention domain scores (0.09, 95% CI: 0.01-0.17, P = 0.01). Peer observations resulted in improvement in the other three domains as well although the improvement did not reach statistical significance (Figure 1). The results were robust to adjustment for age, gender, and years as an attending and did not change after including potential confounders in the model (Figure 1).

Perceptions of the teaching attendings

Of the 49 completed surveys by teaching attendings, 79% reported being “very comfortable” or “somewhat comfortable”, while others were “impartial” and “somewhat uncomfortable” (20%) with being observed by a colleague. Survey participants who reported being uncomfortable provided feedback such as: “I just don’t like being observed”, “I am still a new attending”, or “made me self-aware … and anxious”. The majority of the surveys reported that feedback was “very helpful” or “somewhat helpful” (92%); while 8% were “impartial”. In 90% of the surveys, teaching attendings reported feeling comfortable being observed in the future while in 10% of the surveys, teaching attendings were “not sure”. In the survey, we also asked if teachers had any suggestions to improve the peer observation experience and we received useful feedback (Table 2). Furthermore, teachers listed a number of suggestions they received from their observing peers that they would like to incorporate in their teaching (Table 3).

**Table 2:** Examples of suggestions from teaching and observing hospitalists on how to improve the peer observation process

From Teachers	From Observers
Do it more!	More frequent observations.
Observation should be for the duration of the whole rounds.	Increase clarification of observer’s role to the residents and students.
Learning teaching styles in a different way would be more comfortable.	Some of the "behaviors" on the observation tool are difficult to assess during a 1-hour.
One-hour observations may not enough to see the different styles of interaction.	Deciding with team on best day to observe when most learners on the team are there
The observer should remain innocuous during the observation process	Setting up scheduled time for feedback session
Providing a digital copy of the feedback so it is easier to keep track of	A cheat sheet on main areas of discussion during feedback.
Pre-rounds discussion with the faculty member to talk about what he or she wants to work on in their clinical teaching.	

**Table 3:** Examples of comments by observing and teaching hospitalists on what they learned during a peer observation session

From Teachers	From Observers
Letting residents and students lead the encounter with the patients.	Adding 30-60 second teaching pearls with as many patients as possible. Sprinkling clinical pearls throughout rounds.
Having medical students practice exam skills in a patient with interesting exam findings.	Minimize interruptions. Talking less may have a powerful impact.
Having med students to shorten their presentations while also presenting all the pertinent information.	Starting with students for interpretation of images during rounds.
Moving patient-related conversations to the bedside and in front of patients to allow for more direct observation of resident and students' skills.	Using students as a resource for clinical needs.
Identifying teaching points during rounds.	Being mindful of noting one's own limitations and instances where there is uncertainty and relaying the realities of clinical decision-making.
Quick review EKG and imaging during rounds.	Adding a fun game to rounds, such as "star points" for great presentations, explanations to reasoning, or interactions with patients.
Be more inclusive during discussions at morning rounds among the different learners. Engaging all learners.	Relaxed style on rounds which allow questions and stimulated discussion.
Identifying a particular patient or teaching point prior to rounds for high yield teaching.	Time management so all patients get adequate time based on the complexity of their disease.
Using game-like structure during rounds.	Allowing residents to discuss their plan with each other and come to a decision before "jumping in".
Assign literature search to resident or student during rounds to ensure topics are researched and disseminated to the team. Bring discussion on assigned literature to the bedside using a relevant clinical situation.	Letting senior resident lead the team and being less directive as an attending.
Asking question during rounds from learners rather than jumping in to teach.	Showing respect and thanking learners for presentations/contributions.
Balancing supervision and autonomy was helpful.	Displaying more enthusiasm through mannerisms or voice inflections.
Incorporating literature references.	Combining positive and constructive feedback.
When visiting a patient with contact precautions and students are staying outside the room, give them a short clinical question to look up while you are in the room.	Using more prompts and reflective questions to push learners to think and make connections, rather than asking directive questions.
Staying on a topic and less digression during rounds.	Reviewing teaching points from the previous day on rounds with quick questions.

Perceptions of the peer observer

Of the 33 surveys completed by peer observers, the majority reported that it was “very easy” or “somewhat easy” to complete the peer observation tool (36% and 64%, respectively). In 94% of the surveys, observers found it “very easy” or “somewhat easy” to provide feedback to their peers, and 6% found it “somewhat difficult” to give feedback. Those who struggled to give feedback, elaborated by providing comments such as: “difficult trying to balance constructive feedback with positive feedback”, “little hesitant to give feedback on peer behaviors”, “I was a little nervous”, or “finding time to meet and speak in relatively private area”. The majority of observers rated the value of the observation experience as “very helpful” or “somewhat helpful” (88%); while 12% were “impartial” or found feedback “somewhat unhelpful”. Similar to teachers, observers also listed teaching behaviors that they observed during peer observation and wanted to adopt (Table 2) and provided suggestions on how to improve the peer observation process (Table 3).

## Discussion

In this study, we found that peer observation and feedback resulted in a significant and positive improvement in two of the five teaching domains. The association was independent of potential confounders (age, gender, and years as attending). Although three of the five domains (control of session, evaluation, and feedback) did not reach statistical significance, we observed a trend toward improved teaching behavior in these domains. We show that the peer observation of teaching behaviors is feasible, and relatively short observations are valuable. To the best of our knowledge, this is the first study that has shown a direct and positive association between peer observations with feedback followed by an objective assessment of improvement in teaching behaviors. Further, we developed and tested a peer observation and feedback tool designed for the inpatient setting. Both teaching attendings and peer observers found the peer observation with feedback valuable, which facilitated the project over time and helped to set an expectation that a peer observation would occur weekly. This contributed to a culture of learning among faculty, moving from context-based learning to a community of practice. Observers were able to identify behaviors demonstrated by the teaching attendings that they would like to incorporate into their own practice, which added a dimension of reciprocity to the activity.

While there are several studies supporting the use of the SFDP clinical teaching framework, these studies are limited in their scope and conclusions. Litzelman et al. in 1998 performed a factor analysis on a survey based on the SFDP framework which was administered to students [12]. Their results suggest that there is a correlation between a teacher’s ability to promote self-directed learning and a teacher’s fund of knowledge. In another study, the same authors showed similar results in residents [15]. However, both studies did not utilize the survey among peers, limiting their generalizability to learner assessments. The results may, therefore, be affected by learners’ concerns for their own grades and feedback. Another study by Mookherjee et al. utilized a tool based on the SFDP framework for peer to peer observations among hospitalists, which resulted in increased confidence giving and receiving feedback, as well as increased confidence teaching on rounds [6]. However, they did not evaluate the tool’s effectiveness in promoting change in teaching behaviors. Pierce et al. demonstrated a change in self-reported teaching behaviors after a peer observation of teaching on rounds and feedback discussion, but did not objectively observe a change in skill or teaching behavior [5]. Skeff demonstrated a change in videotaped teaching behaviors after clinician-educators attended a teaching seminar, although the change observed was small and the portion of rounds analyzed was limited [11].

The implications of this study are potentially significant. Based on the experience of other educators who have performed peer observations, observations should be done in a collaborative fashion [16]. They note the effectiveness of “shared empathy” which can occur among peers who have similar experiences. In our study, observers and teachers worked together within the same hospitalist group and had similar clinical and teaching duties. Another key to success noted by Siddiqui et al. is that learning from the experience is optimized when both teachers and observers are comfortable with the experience [16]. Our study found that the majority of both observers and teachers were comfortable with the peer observation process which may have contributed to the improvement in teaching behaviors. At the teacher level, direct observation using a standardized observation tool based on the SFDP framework provided a venue for faculty to receive verbal and written feedback of desired observable teaching behaviors, leading to timely adoption of desired teaching behaviors. Observations serve as a reminder of the behaviors deemed important by the SFDP framework. Peer observations reinforce attitudes regarding the importance of these behaviors as teaching attendings. For the observers, direct peer observation can lead to improvement in their own teaching skills [4,5,8]. Peer observations helped create a shared mental model between the observers and the teachers of what it means to be a good clinical teacher [8]. This peer observation process can be useful for both senior and junior faculty members [7].

At the institution level, there are significant implications regarding faculty development. Our peer observation and feedback process may represent a paradigm shift in faculty development programs. A traditional faculty development program typically occurs outside of the workplace and/or trainee learning environment and may not fully address the challenges of translating learned behaviors into the workplace [17]. Our peer observations occur within the clinical teaching environment and allows for the development of clinically applicable teaching techniques that can be shared among colleagues. In addition, the effectiveness of the individualized formative peer feedback and subsequent faculty development that occurs is related to the concrete, objective, and timely feedback that is provided from credible colleagues over different encounters [18,19]. Other studies have shown that peer observation feedback can be shared amongst faculty groups leading to quick adoption of specific teaching behaviors [5]. Faculty engagement and motivation for change is key to successful faculty development and has been cited as a common barrier to participation in faculty development programs amongst clinical teachers [20]. We saw positive results in less than a year of peer observations; therefore, our peer observation process may allow for a rapid and consistent development of faculty, especially junior faculty [21].

This study has several strengths and potential limitations. We were able to limit observer bias by avoiding the same observer-teacher pairings for follow-up observations. Furthermore, our study’s endpoint, observed teaching behaviors, was objective compared to other studies. The observation tool allows objectivity in that the observer can note whether behaviors were observed or not observed. A potential limitation of the observation tool is that it cannot differentiate whether these behaviors were not observed because the teacher did not perform them or because the opportunity to see such behaviors did not exist. It is possible that teachers may have changed their behavior due to their awareness of being directly observed (Hawthorne effect), which is a limitation of any observational study. Furthermore, this effect may be diminished over multiple observations. While we did not study long-term feasibility of peer observation, the observers’ time commitment of an hour to observe the teacher plus time to complete evaluation forms and questionnaires and schedule a dedicated verbal feedback session may impact its long-term feasibility. The cost to the institution of a peer observation program will be variable and will depend on how it is implemented.

## Conclusions

In summary, we have shown that peer observation of bedside teaching using a standardized observation tool provides timely and valuable feedback to faculty on their clinical bedside teaching sessions. We observed an increase in the occurrence of desirable teaching behaviors. Moreover, the observations were well-received by the faculty. Further research in improving teaching skills and incorporation of best teaching practices during clinical rounds will help to improve bedside clinical teaching of learners.
